# Regulation of Acetylcholine Quantal Release by Coupled Thrombin/BDNF Signaling in Mouse Motor Synapses

**DOI:** 10.3390/cells8070762

**Published:** 2019-07-22

**Authors:** Alexander Gaydukov, Polina Bogacheva, Ekaterina Tarasova, Anastasia Molchanova, Anna Miteva, Ekaterina Pravdivceva, Olga Balezina

**Affiliations:** Department of Human & Animal Physiology, Lomonosov Moscow State University, Leninskie Gory 1/12, 119991 Moscow, Russia

**Keywords:** endplate potentials, neuromuscular junction, thrombin, BDNF, TrkB, MEK1/2, A_2A_ receptors, PKA, PLC

## Abstract

The aim of this study was to compare the acute effects of thrombin and brain-derived neurotrophic factor (BDNF) on spontaneous miniature endplate potentials (MEPPs) and multiquantal evoked endplate potentials (EPPs) in mouse neuromuscular junctions (NMJs) of m. diaphragma and m. EDL. Intracellular microelectrode recordings of MEPPs and EPPs were used to evaluate the changes in acetylcholine (ACh) release in mature and newly-formed mouse NMJs. Thrombin (1 nM) increased the amplitude of MEPPs and EPPs by 25–30% in mature and newly-formed NMJs. This effect was due to an enhanced loading of synaptic vesicles with ACh and increase of ACh quantal size, since it was fully prevented by blocking of vesicular ACh transporter. It was also prevented by tropomyosin-related kinase B (TrkB) receptors inhibitor ANA12. Exogenous BDNF (1 nM) mimicked thrombin effect and increased the amplitude of MEPPs and EPPs by 25–30%. It required involvement of protein kinase A (PKA) and mitogen-activated protein kinase (MEK1/2)-mediated pathway, but not phospholipase C (PLC). Blocking A_2A_ adenosine receptors by ZM241385 abolished the effect of BDNF, whereas additional stimulation of A_2A_ receptors by CGS21680 increased MEPP amplitudes, which was prevented by MEK1/2 inhibitor U0126. At mature NMJs, BDNF enhanced MEPPs frequency by 30–40%. This effect was selectively prevented by inhibition of PLC, but not PKA or MEK1/2. It is suggested that interrelated effects of thrombin/BDNF in mature and newly-formed NMJs are realized via enhancement of vesicular ACh transport and quantal size increase. BDNF-induced potentiation of synaptic transmission involves the functional coupling between A_2A_ receptor-dependent active PKA and neurotrophin-triggered MAPK pathway, as well as PLC-dependent increase in frequency of MEPPs.

## 1. Introduction

In recent years, there has been a growing interest in muscle-derived factors—myokines, and their physiological activities. Myokines are a family of substances variable in structure, which can be released from skeletal muscle fibers and produce multiple systemic or local effects in organs and tissues [1,2,3,4]. A well-known neurotrophin BDNF has been recently rediscovered as a myokine [2,4,5]. Indeed, the expression of proBDNF [6,7] and its release from skeletal muscle with subsequent regulatory action of its mature form on muscle receptors and metabolism was shown [5]. Skeletal muscle fibers may serve as a source not only of a growing number of myokines, but also of active thrombin [8,9]. Prothrombin expression in skeletal muscle was reported already [10]. Being released from neonatal muscles or during the reinnervation of mature muscle fibers, thrombin can activate muscular PAR1 receptors, inducing structural and functional plasticity of NMJs [11,12,13]. The particular signaling pathways triggered by thrombin and/or BDNF in motor synapses, remain poorly understood. Recently, we have found that activation of thrombin receptors of PAR1-type by their peptide agonist TRAP6 (SFLLRN, 1 µM) leads to a fast and sustained increase in the amplitude of MEPPs at both mature and newly-formed motor synapses of mice [14]. Despite the lack of PAR1 receptors at the presynaptic membrane in postnatal motor synapses [15], TRAP6-induced increase in MEPP amplitude seemed to be of presynaptic origin since it was prevented by vesamicol—an inhibitor of vesicular ACh transporter. Moreover, this PAR1 agonist-mediated effect was also prevented by blocking TrkB receptors [14]. TrkB receptors are localized pre- and postsynaptically at NMJs and serve as targets for neurotrophins including BDNF [16]. We have found, that exogenously applied BDNF (1 nM) caused an increase in MEPPs amplitude, similar to TRAP6 [14]. We assumed that the similarity of the potentiating effects between TRAP6 and BDNF may reflect a complex chain of events starting with the activation of postsynaptic PAR1 receptors in the muscle, thus stimulating the release of myogenic BDNF. In turn, BDNF, acting retrogradely on TrkB receptors, localized on motor nerve terminals, may trigger some signaling pathway stimulating ACh loading into synaptic vesicles, thus increasing MEPP amplitude presynaptically. To test this hypothesis, it was necessary to reveal if thrombin may act similarly to TRAP6, increasing the amplitude of postsynaptic potentials, and to confirm the assumption that presynaptic action of BDNF can stimulate the vesicular ACh transport, thereby leading to an increase in MEPP amplitude via enlargement of ACh quantal size. The aim of the present study was to clarify these issues. We compared the acute effects of exogenously applied thrombin and BDNF on the amplitude of postsynaptic endplate potentials in mature and newly-formed mouse NMJs. Further, we investigated the ability of BDNF to trigger different signaling pathways in nerve terminals that can induce the increase of ACh transport into synaptic vesicles or provoke some other effects in mouse NMJs.

## 2. Materials and Methods

### 2.1. Animals and Neuromuscular Preparations

Experiments were performed on isolated extensor digitorum longus muscle (m. EDL) and hemidiaphragm neuromuscular preparations of adult (weighing 25–30 g, 7–8 weeks old) mice (strain BALB/c) of either sex.

Intact neuromuscular preparations were used when solely spontaneous synaptic activity (without nerve stimulation) was studied. To record synaptic activity evoked by nerve stimulation, cut neuromuscular preparations of m. EDL or hemidiaphragm were used to prevent contraction as well as to record both spontaneous and evoked endplate potentials from the same synapse [17]. Immediately after the transverse cutting of muscle fibers the preparation was thoroughly washed in a large volume (more than 150 mL) of Liley solution (see below) for more than 1 h to prevent the action potential conduction block. As a result, the recorded value of the resting membrane potential (RMP) was lower in cut fibers (<−50 mV) than in intact ones.

Synaptic transmission in newly-formed neuromuscular junctions was studied in reinnervated m. EDL of mice. To induce reinnervation process and synaptic formation, surgical denervation of m. EDL was performed. Mice were anesthetized with 5% isoflurane and a small segment (1 mm) of the left peroneal nerve was aseptically crushed approximately 10 mm from its entrance into the muscle. The wound was sutured and animals were allowed to recover for 11 days. It was shown that after such procedure, functional reinnervation starts at 8–9 days after nerve crush [18]. Spontaneous synaptic transmission at the newly-formed junctions is characterized by low frequency and prolonged time course of MEPPs compared to functionally mature synapses. Amplitude distribution of MEPPs is also altered and doesn’t fit to normal curve due to appearance of both low amplitude events and so called “giant miniature” events [18]. Evoked endplate potentials of newly-formed NMJs demonstrate lower amplitude and quantal content, prolonged time course and longer synaptic delay [19]. During repetitive firing no depression of transmission is observed throughout a short train of stimuli (50 Hz, 1 s), but instead a facilitation that reaches a stable level that is higher than first EPP in train [20,21].

All animal handling and experimental procedures were performed in full accordance with the EC guidelines (Directive 86/609/EEC on the treatment of laboratory animals). Mice were housed under a 12-h light/dark cycle with free access to food and water. The experimental protocols were approved by the Bioethics committee of the MSU Biological department (protocol number 2018-02-22-88-0-2). Mice were sacrificed by quick decapitation.

### 2.2. Electrophysiology

The m. EDL with supplied nerve or left hemidiaphragm with attached phrenic nerve were excised and stretched in an experimental chamber superfused (0.5 mL/min) by oxygenated (95% O_2_, 5% CO_2_) Liley solution (pH 7.2–7.4) containing the following (in mM): KCl, 4; NaCl, 135; NaH_2_PO_4_, 0.9; MgCl_2_, 1; CaCl_2_, 2; NaHCO_3_, 16.3; glucose, 11. All experiments were performed at room temperature (20–22 °C). MEPPs and EPPs were recorded intracellularly using glass microelectrodes filled with 2.5 M KCl (tip resistance was 10–20 MΩ) and connected to a Neuroprobe Amplifier Model 1600 (A-M Systems, Sequim, WA, USA) or Axoclamp-2B (Molecular Devices, San Jose, CA, USA). The signals were digitized using an analog-digital converter E-154 (L-Card, Moscow, Russia) with a PowerGraph 6.0 interface and analyzed using MiniAnalysis software (Synaptosoft, Decatur, GA, USA). MEPPs were recorded for 120 s in cases of studying spontaneous activity of NMJs. To study evoked synaptic activity, the peroneal nerve or phrenic nerve was stimulated by short (1 s) high-frequency (50 Hz) trains of suprathreshold pulses (the duration of each pulse was 0.08–0.1 ms). To avoid fatigue of motor synapses and consequent amplitude rundown and changes of the EPP pattern which were not influenced by the drugs added to perfusion system, there were pauses of 4–5 min between stimulations of nerve. Before starting the stimulation of nerve, in each synapse MEPPs were recorded for 100 s. Mean value of the MEPP amplitudes recorded within this period was used for calculation of the EPP quantal content. MEPPs and EPPs for at least five different synapses were recorded in control. After control recordings, tested drugs were added to the perfusion solution in the indicated order, and the activity of various synapses was recorded within 45 min–1.5 h of drugs application. In each experimental series, no fewer than three neuromuscular preparations were used.

### 2.3. Drugs

The following drugs were used: Thrombin from human plasma (purchased from Sigma-Aldrich, USA); human isoform of BDNF (purchased from Alomone Labs, Jerusalem, Israel); (±)-Vesamicol hydrochloride and Bafilomycin A1 as direct and indirect inhibitor of vesicular ACh transport, respectively; ANA12 as TrkB receptor antagonist, U73122 and U73343 as PLC inhibitor and its inactive analog, respectively; U0126 and U0124 as MEK1/2 inhibitor and its inactive analog, respectively; H-89 dihydrochloride as PKA inhibitor; ZM241385 as adenosine A_2A_ receptor antagonist and CGS21680 as A_2A_ receptor agonist (purchased from Tocris, Bio-Techne, Minneapolis, MN, USA). Thrombin and BDNF were dissolved in deionized water. Stock solutions of all other drugs were prepared in DMSO (Helicon, Moscow, Russia). The final concentrations of DMSO in the working solution did not exceed 0.01% (*v*/*v*). At this concentration, the solvent did not affect the parameters of spontaneous and evoked activity in mouse NMJs. All drugs were applied via bath perfusion system (0.5 mL/min).

### 2.4. Data Processing and Statistical Analysis

The values of muscle fiber RMP, the amplitude and time course of MEPPs and EPPs, and the MEPP frequency were estimated. When only spontaneous synaptic activity was studied using intact neuromuscular preparations, the amplitudes of MEPPs were normalized to −70 mV to correct for the changes in the driving force caused by the voltage shift upon the RMP changes. In cut neuromuscular preparations, the amplitudes of MEPPs and EPPs were first normalized to the membrane potential of −50 mV [22]. The quantal content of EPPs was calculated as the ratio between the mean normalized EPP amplitude corrected for nonlinear summation [23] and the mean normalized amplitude of MEPPs. Statistical analysis was performed using GraphPad Prism 6.0 software (GraphPad Software, San Diego, CA, USA). With the exception of the representative original recordings and cumulative probability curves, all data in the text and figures are presented as the means ± standard error of the means; n corresponds to the number of synapses in the group. The normality of the parameter distribution was verified by the D’Agostino–Pearson normality test. The significance between two compared groups was estimated by unpaired Student’s *t*-test when the distributions were normal and the Mann–Whitney rank sum test when the distribution was not normal. The Kholmogorov-Smirnov test was used when cumulative probabilities of MEPP amplitudes were analyzed. Two-way ANOVA (with the post hoc Bonferroni correction) was used for the analysis of EPP amplitude and quantal content. Values of *p* < 0.05 were considered statistically significant.

## 3. Results

**Figure 1:**
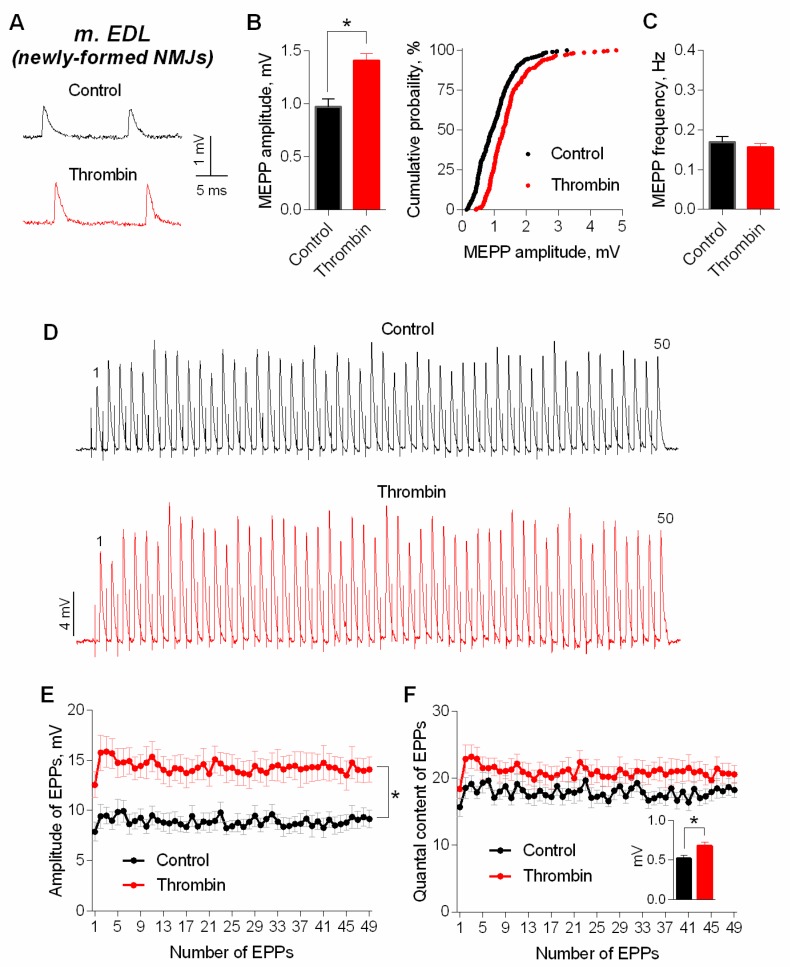
Thrombin in low concentration (1 nM) increases amplitudes of spontaneous and evoked postsynaptic endplate potentials at newly-formed NMJs of reinnervated mouse m. EDL. (**A**) Representative recordings of MEPPs in control (above) and upon application of thrombin (below). (**B**) Mean amplitude of MEPPs (left) and cumulative probability plots (right) in control (*n* = 29) and upon application of thrombin (*n* = 31). (**C**) Mean frequency of MEPPs in control and in the presence of thrombin. (**D**) Representative recordings of EPPs during a short (1 s) high-frequency (50 Hz) train in control (above) and upon application of thrombin (below). (**E**) Changes in the EPP amplitude in control (*n* = 16) and in the presence of thrombin (*n* = 16). (**F**) Quantal content of EPPs in control and upon application of thrombin. Inset shows MEPP amplitudes. Histograms, symbols (except the ones in cumulative probability plots) and error bars represent the mean ± SEM. * *p* < 0.05 compared to control.

First, we analyzed the effect of exogenously applied thrombin (1nM) on spontaneous and evoked activity of mouse NMJs which were newly-formed during the reinnervation of m. EDL 11 days after the motor nerve crush. In presence of thrombin we did not reveal any changes in RMP (−66.5 ± 1.0 mV in control (*n* = 29) and −65.5 ± 0.7 mV under thrombin (*n* = 31, *p* > 0.05). Time parameters of MEPPs were unchanged either: rise time was 2.10 ± 0.10 ms in control and 2.18 ± 0.10 ms under thrombin (*p* > 0.05); half-decay was 4.58 ± 0.17 ms in control and 4.71 ± 0.21 ms under thrombin (*p* > 0.05). Thrombin did not cause any significant changes in MEPP frequency, but the amplitude of MEPPs increased significantly by an average of 25–30%, and cumulative probability curves of MEPP amplitudes distribution shifted uniformly towards higher values in presence of thrombin (Figure 1A–C).

Next, we cut neuromuscular preparations to test the action of thrombin on evoked ACh release during short, rhythmical EPP trains (50 Hz, 1 s) in newly-formed synapses of m. EDL. Thrombin caused a uniform increase in amplitude of EPPs throughout the train on average, by 30% (Figure 1D,E). The amplitude of MEPPs, recorded simultaneously in the same synapses where registration of EPPs was performed, changed in the same way. The analysis of EPPs quantal content revealed that the increase in the amplitude of multiquantal EPPs was not due to increase in EPP quantal content (Figure 1F).

**Figure 2:**
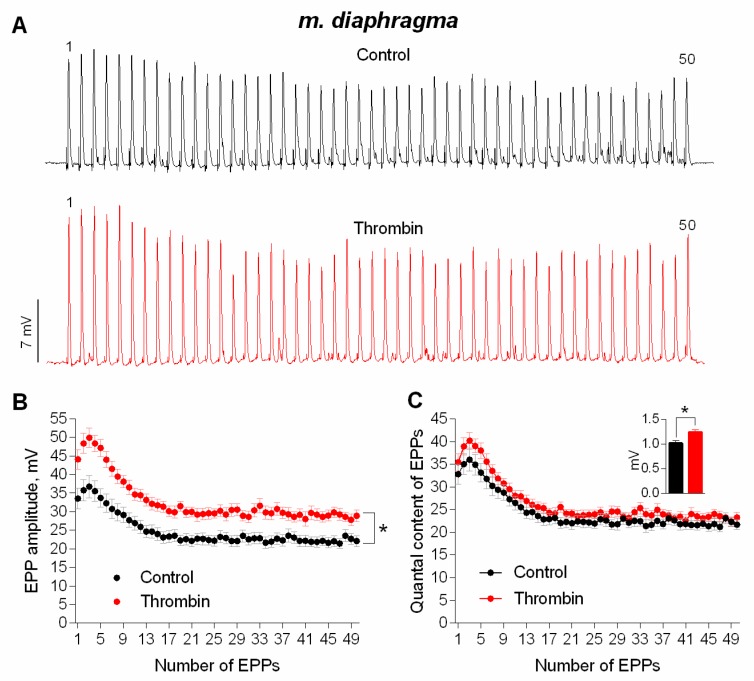
Thrombin-induced upregulation of evoked neuromuscular transmission during short (1 s) high-frequency (50 Hz) trains in mouse diaphragm. (**A**) Representative recordings of EPPs in control (above) and upon application of thrombin (1 nM) (below). (**B**) Changes in the EPP amplitude in control (*n* = 17) and upon application of thrombin (*n* = 17). (**C**) Quantal content of EPPs in control and upon application of thrombin. Inset shows MEPP amplitudes. Histograms, symbols and error bars represent the mean ± SEM. * *p* < 0.05 compared to control.

It was necessary to investigate the ability of thrombin to exert a similar potentiating effect on synaptic transmission in mature NMJs of diaphragm. As well as in the newly-formed synapses of m. EDL, thrombin uniformly potentiated the amplitude of each EPP during the short rhythmic train (50 Hz, 1 s) in mature NMJs (Figure 2A,B). The increase in EPPs amplitude was accompanied by a comparable increase in the amplitude of MEPPs recorded in the same synapses, thereby leaving the value of EPP quantal content unchanged in presence of thrombin compared to control (Figure 2C).

**Figure 3:**
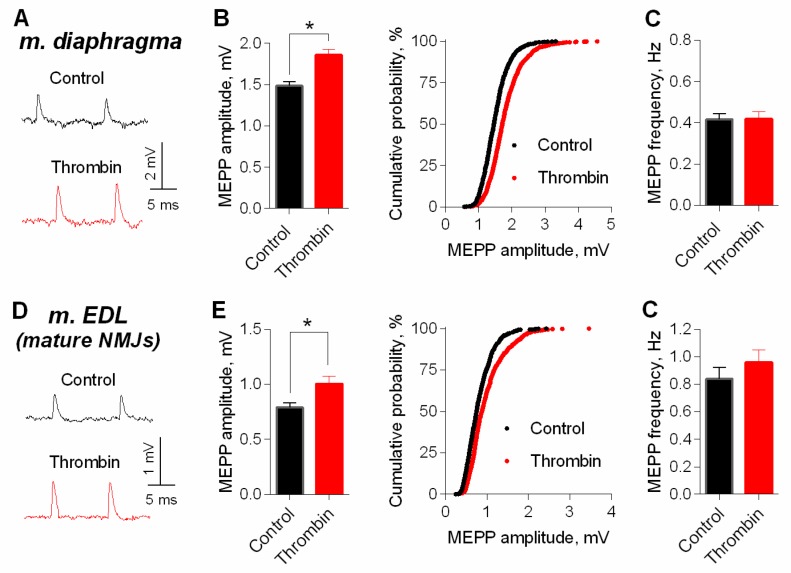
Thrombin-induced potentiation of spontaneous ACh release at mature NMJs. (**A**) Representative recordings of MEPPs in diaphragm in control (above) and upon application of thrombin (1 nM) (below). (**B**) Mean amplitude of MEPPs (left) and cumulative probability plots (right) in control (*n* = 24) and upon application of thrombin (*n* = 32). (**C**) Mean frequency of MEPPs in control and in the presence of thrombin. (**D**) Representative recordings of MEPPs in m. EDL in control (above) and upon application of thrombin (1 nM) (below). (**E**) Mean amplitude of MEPPs (left) and cumulative probability plots (right) in control (*n* = 28) and upon application of thrombin (*n* = 31). (**F**) Mean frequency of MEPPs in control and in the presence of thrombin. Histograms and error bars represent the mean ± SEM. * *p* < 0.05 compared to control.

Next, we used intact neuromuscular preparations (with non-cut muscle fibers) for a more detailed electrophysiological analysis of thrombin effect on MEPPs amplitude. The application of thrombin led to an increase in the amplitude of MEPPs in mature NMJs of both m. EDL and diaphragm. Thrombin caused a shift of cumulative probability curves of MEPP amplitudes distribution in NMJs of both muscle types to the right (Figure 3).

**Figure 4:**
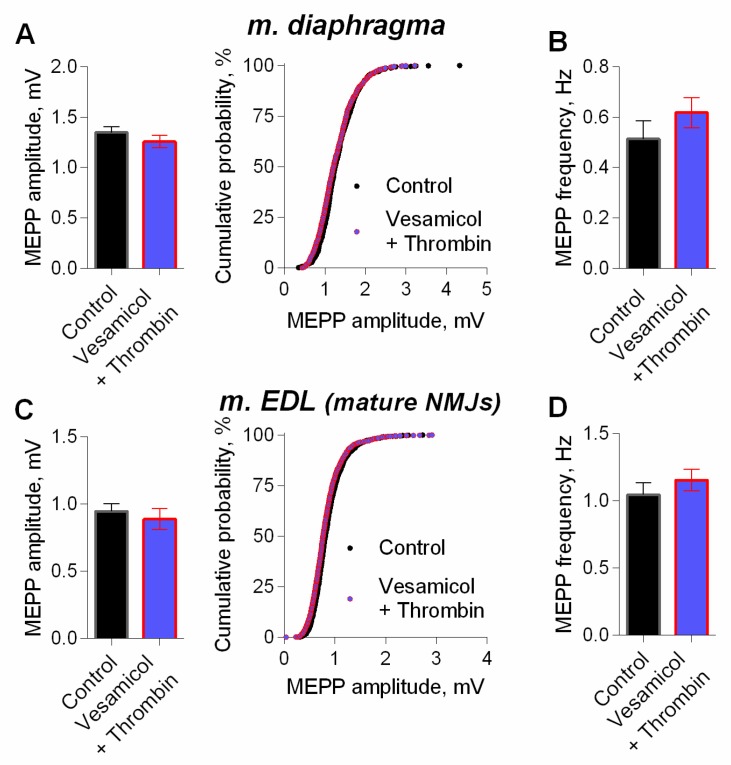
Inhibition of vesicular ACh transporter abolished thrombin-induced increase in MEPP amplitude. (**A**) Mean amplitude of MEPPs (left) and cumulative probability plots (right) in control (*n* = 21) and upon application of thrombin (1 nM) in the presence of vesamicol (1 μM) (*n* = 24) at diaphragm NMJs. (**B**) Mean frequency of MEPPs in control and upon application of thrombin in the presence of vesamicol at diaphragm NMJs. (**C**) Mean amplitude of MEPPs (left) and cumulative probability plots (right) in control (*n* = 25) and upon application of thrombin in the presence of vesamicol at mature NMJs of m. EDL (*n* = 28). (**D**) Mean frequency of MEPPs in control and upon application of thrombin in the presence of vesamicol at m. EDL mature NMJs. Histograms and error bars represent the mean ± SEM.

We further examined whether inhibitor of vesicular ACh transporter—vesamicol—may counteract the effect of thrombin. We have previously shown that vesamicol (1 μM) has no significant effect of on MEPP amplitude at NMJs neither in m. EDL [24] nor in diaphragm [14,25]. However, in the presence of vesamicol, thrombin completely lost its ability to increase MEPP amplitude at NMJs of both muscle types (Figure 4). Taken together, these results suggest that an increase in the size of each quantum of ACh in the multiquantal EPP was the main cause of thrombin-mediated increase in EPPs amplitudes. Thus, we confirmed that in low, nanomolar concentration thrombin acts like PAR1 peptide agonist in micromolar concentration [14], and is able to induce an increase in MEPPs and EPPs amplitudes. This acute effect of thrombin on neuromuscular synaptic transmission seems to be related to the enhancement of ACh transport into synaptic vesicles in motor nerve terminals, since it was blocked by vesamicol. We suggest that the upregulation of vesicular transport of ACh is the final part in the signaling cascade triggered by thrombin activation of postsynaptic PAR1 receptors. Given the absence of PAR1 on the presynaptic membranes of mature and juvenile NMJs [12,15], the most likely conclusion is that thrombin, acting postsynaptically, induces the release of a retrogradely acting muscle-derived factor into the synaptic cleft, that triggers the increase of vesicular ACh loading on presynaptic level. Taking into account our previous findings [14], we hypothesized that myogenic BDNF may play the role of such retrogradely acting factor.

**Figure 5:**
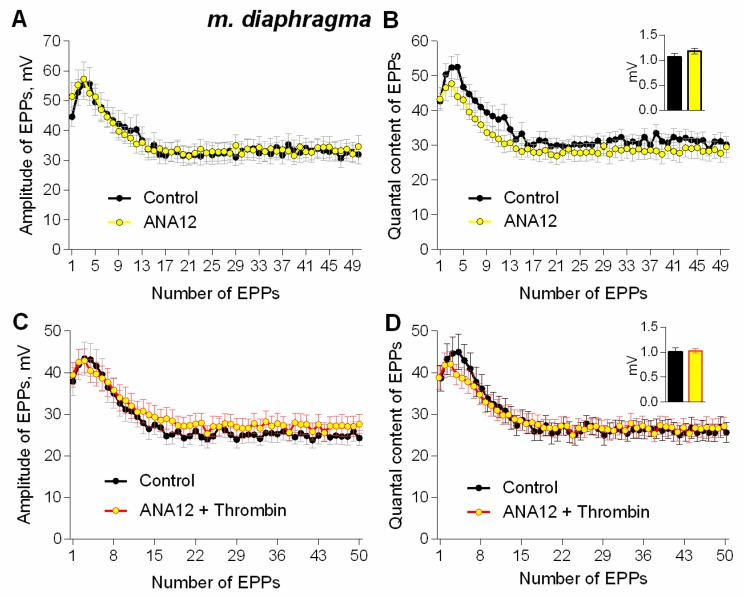
TrkB receptors mediate thrombin-induced upregulation of evoked neuromuscular transmission during short (1 s) high-frequency (50 Hz) trains at NMJs in mouse diaphragm. (**A**) Changes in the EPP amplitude in control (*n* = 17) and upon application of TrkB receptor antagonist ANA12 (10 μM) (*n* = 24). (**B**) Quantal content of EPPs in control and upon application of ANA12. (**C**) Changes in the EPP amplitude in control (*n* = 17) and upon application of thrombin (1 nM) (*n* = 16) in the presence of ANA12 (*n* = 23). (**D**) Quantal content of EPPs in control and upon application of thrombin in the presence of ANA12. Histograms, symbols and error bars represent the mean ± SEM. The insets show MEPP amplitudes.

**Figure 6:**
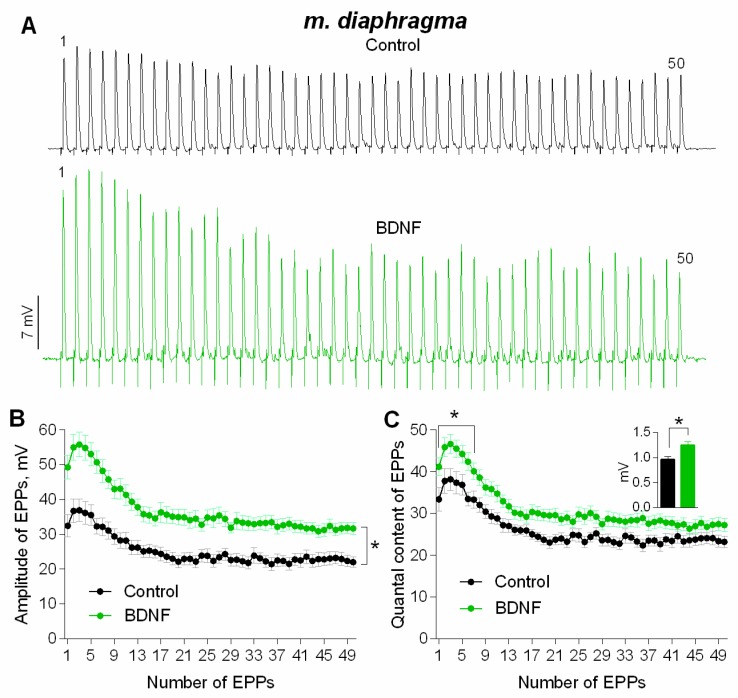
BDNF-induced potentiation of evoked neuromuscular transmission during short (1 s) high-frequency (50 Hz) trains at diaphragm NMJs (**A**) Representative recordings of EPPs in control (above) and after BDNF (1 nM) was added (below). (**B**) Changes in the EPP amplitude in control (*n* = 20) and upon application of BDNF (*n* = 23). (**C**) Quantal content of EPPs in control and upon application of BDNF. Inset shows MEPP amplitudes. Histograms, symbols and error bars represent the mean ± SEM. * *p* < 0.05 compared to control.

To test this hypothesis, we examined the possible involvement of TrkB receptors in thrombin-induced potentiation of synaptic transmission at mature NMJs of mouse diaphragm. TrkB antagonist ANA12 (10 μM) did not significantly change the amplitude or quantal content of EPPs in short rhythmic trains (50 Hz, 1 s) (Figure 5A,B), which is consistent with previous reports [14,26]. However, in the presence of ANA12, thrombin failed to increase the amplitude of the first and subsequent EPPs in the train (Figure 5C,D). These data suggest that thrombin-induced potentiation of neuromuscular synaptic transmission depends on the activation of TrkB receptors. Therefore, we further investigated whether the activation of these receptors by exogenous BDNF can mimic the effects of thrombin at NMJs. Application of exogenous BDNF (1 nM) to mature NMJs of diaphragm muscle increased the amplitude of EPPs in trains on average by 30%. Similar to the thrombin-induced potentiation of evoked ACh release, BDNF-triggered increase in EPP amplitude was accompanied by a comparable increase in MEPP amplitude (Figure 6). In contrast to the lack of any changes of EPP quantal content when thrombin was applied to NMJs, BDNF caused a statistically significant increase in quantal content of EPPs by 20% at the initial stage of the EPP train (Figure 6C). Concerning the more complex effect of BDNF than the one of thrombin on evoked synaptic transmission, we used intact neuromuscular preparations for a more detailed electrophysiological study of the effect of this neurotrophin on spontaneous ACh secretion in mature and newly-formed NMJs.

**Figure 7:**
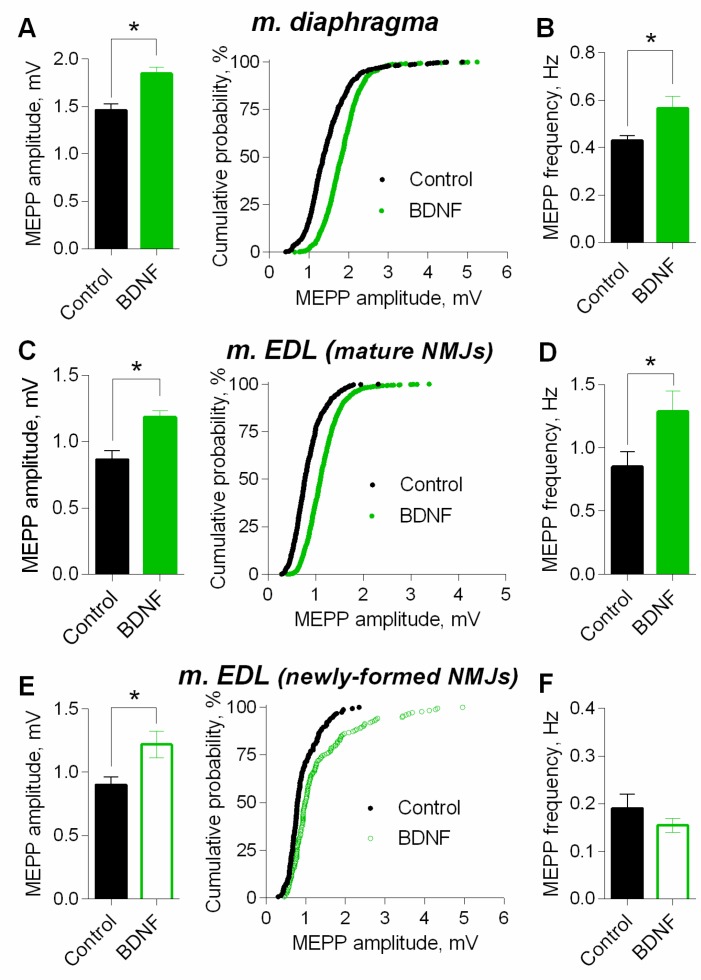
BDNF (1 nM) potentiates spontaneous ACh release at mouse NMJs. (**A**) Mean amplitude of MEPPs (left) and cumulative probability plots (right) in control (*n* = 21) and upon application of BDNF (*n* = 22) at diaphragm NMJs. (**B**) Mean frequency of MEPPs in control and in the presence of BDNF a diaphragm NMJs. (**C**) Mean amplitude of MEPPs (left) and cumulative probability plots (right) in control (*n* = 15) and upon application of BDNF (*n* = 21) at mature NMJs in m. EDL. (**D**) Mean frequency of MEPPs in control and after BDNF was applied at mature NMJs in m. EDL. (**E**) Mean amplitude of MEPPs (left) and cumulative probability plots (right) in control (*n* = 15) and upon application of BDNF (*n* = 19) at newly-formed NMJs in reinnervated m. EDL. (**F**) Mean frequency of MEPPs in control and after BDNF was applied at newly-formed NMJs in reinnervated m. EDL. Histograms and error bars represent the mean ± SEM. * *p* < 0.05 compared to control.

At mature NMJs in both diaphragm and m. EDL, BDNF (1nM) caused an increase in MEPP amplitude by 25–30%. Moreover, the application of exogenous neurotrophin also provoked a significant increase in MEPP frequency at both types of muscles used (Figure 7A–D). However, at newly-formed NMJs of reinnervated m. EDL BDNF potentiated MEPP amplitude by the same extent as in mature motor synapses but failed to increase the MEPP frequency (Figure 7E,F).

**Figure 8:**
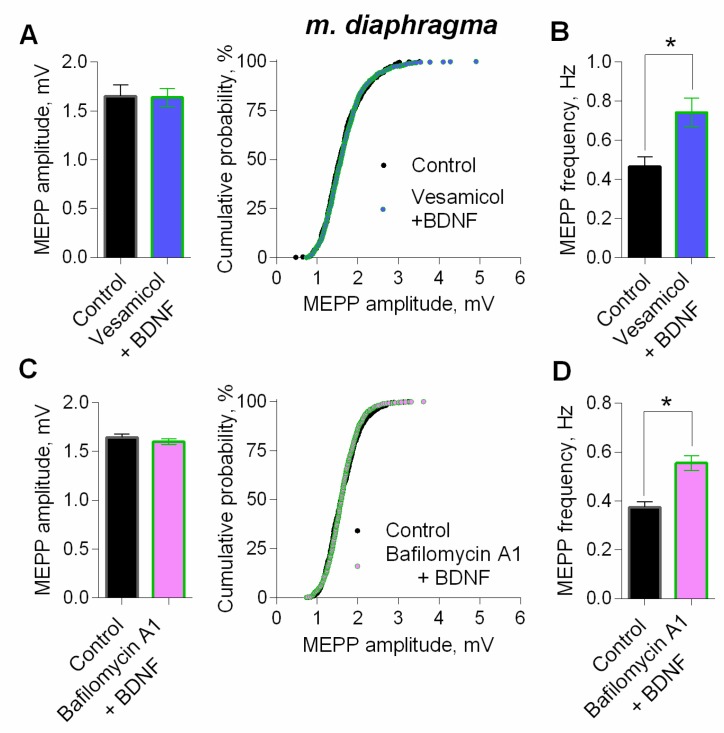
BDNF (1 nM) increases MEPP amplitude at the mouse diaphragm NMJs due to the enlargement of ACh quantal size. (**A**) Mean amplitude of MEPPs (left) and cumulative probability plots (right) in control (*n* = 15) and after BDNF was added in the presence of preapplied vesamicol (1 μM) (*n* = 17). (**B**) Mean frequency of MEPPs in control and upon application of BDNF in the presence of vesamicol at diaphragm NMJs. (**C**) Mean amplitude of MEPPs (left) and cumulative probability plots (right) in control (*n* = 19) and after BDNF was added in the presence of preapplied bafilomycin A1 (0.3 μM) (*n* = 27). (**D**) Mean frequency of MEPPs in control and upon application of BDNF in the presence of bafilomycin A1. Histograms and error bars represent the mean ± SEM. * *p* < 0.05 compared to control.

Next, we studied the signaling mechanisms that determine the potentiating action of BDNF on parameters of spontaneous ACh release in motor synapses, using mature diaphragm NMJs. We found that an increase in the MEPP amplitude, as well as a right-shift of cumulative probability curves of the MEPP amplitudes distribution induced by BDNF could be prevented either by direct blocking of vesicular ACh transporter by vesamicol (1 µM), or by bafilomycin A1 (0.3 µM), an inhibitor of v-ATPase, which controls the synaptic vesicles loading with ACh (Figure 8). Neither vesamicol nor bafilomycin A1 at given concentrations do not induce any changes in the parameters of spontaneous ACh secretion at NMJs [25,27,28]. Taken together, the obtained data allowed us to suggest that the increase in the amplitude of the MEPPs (and the EPPs) could be due to the presynaptic effect of BDNF, which triggers intracellular signaling pathways, leading to an increased loading of ACh into synaptic vesicles. It is noteworthy that inhibition of vesicular ACh transport (either direct or indirect) did not prevent the BDNF-induced increase of MEPP frequency (Figure 8B,D).

Therefore, we investigated which signaling pathways triggered by BDNF could be associated with an increase in the amplitude of MEPPs or their frequency.

**Figure 9:**
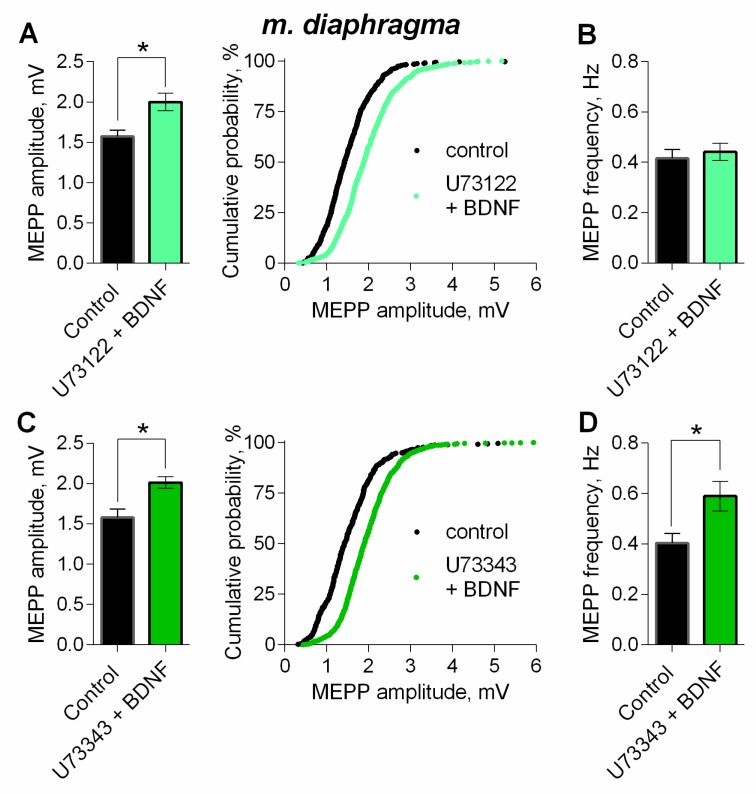
Intracellular signaling mechanisms underlying BDNF-induced upregulation of spontaneous neuromuscular transmission at the mouse diaphragm NMJs. PLC mediates BDNF-induced increase in MEPP frequency. (**A**) Mean amplitude of MEPPs (left) and cumulative probability plots (right) in control (*n* = 25) and after BDNF was added in the presence of PLC inhibitor U73122 (5 μM) (*n* = 28). (**B**) Mean frequency of MEPPs in control and upon application of BDNF in the presence of U73122. (**C**) Mean amplitude of MEPPs (left) and cumulative probability plots (right) in control (*n* = 20) and after BDNF was added in the presence of preapplied inactive analog of U73122 (U73343, 5 μM) (*n* = 27). (**D**) Mean frequency of MEPPs in control and upon application of BDNF in the presence of U73343. Histograms and error bars represent the mean ± SEM. * *p* < 0.05 compared to control.

One of the canonical signaling pathways triggered by BDNF is the phosphoinositide pathway, which starts with TrkB-mediated activation of PLC [29,30,31]. Previously we have shown that PLC inhibitor U73122 (5 µM) caused no significant effect on spontaneous ACh release [14]. However, in the presence of U73122 (5 µM) BDNF failed to increase MEPP frequency, but retained its ability to significantly increase MEPP amplitude (Figure 9A,B). The inactive analogue of the PLC inhibitor, U73343 (5 µM), used as a negative control, proved to be unable to prevent an increase in both amplitude and frequency of MEPPs (Figure 9C,D). These data suggest that presynaptic PLC is not involved in the BDNF-triggered modulation of MEPP amplitudes in motor synapses.

**Figure 10:**
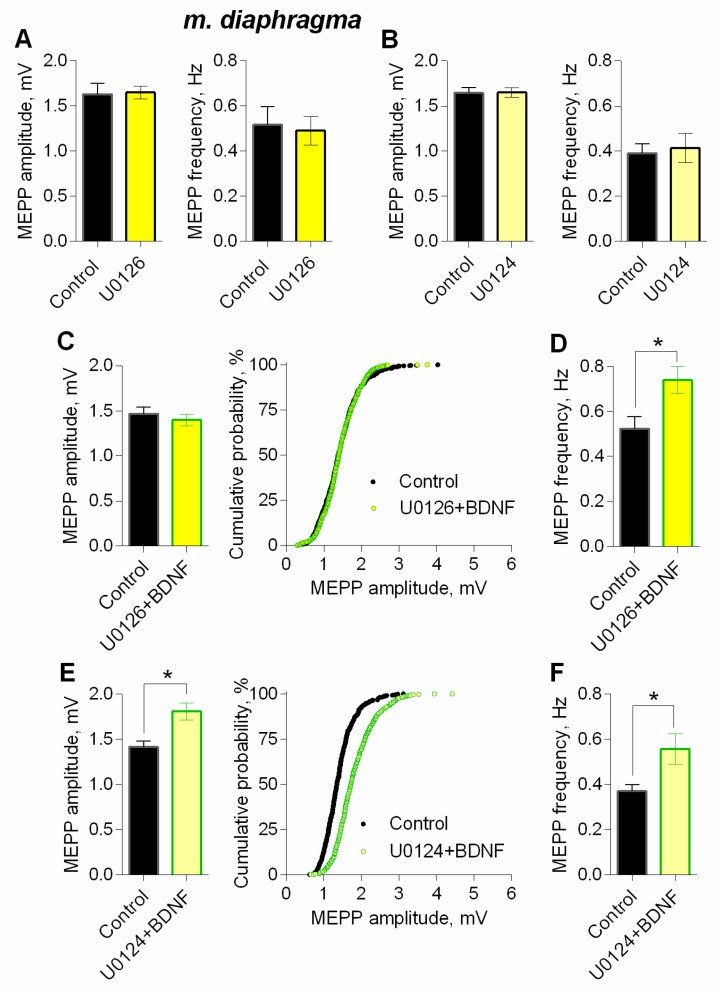
Intracellular signaling mechanisms underlying BDNF-induced upregulation of spontaneous neuromuscular transmission at the mouse diaphragm NMJs. MEK1/2 mediates BDNF-induced increase in MEPP amplitude. (**A**) Mean amplitude of MEPPs (left) and mean MEPP frequency (right) in control (*n* = 16) and upon application of MEK1/2 inhibitor U0126 (20 μM) (*n* = 23). (**B**) Mean amplitude of MEPPs (left) and mean MEPP frequency (right) in control (*n* = 20) and upon application of inactive analog of U0126 (U0124, 20 μM) (*n* = 22). (**C**) Mean amplitude of MEPPs (left) and cumulative probability plots (right) in control (*n* = 24) and after BDNF was added in the presence of preapplied U0126 (*n* = 27). (**D**) Mean frequency of MEPPs in control and upon application of BDNF in the presence of U0126. (**E**) Mean amplitude of MEPPs (left) and cumulative probability plots (right) in control (*n* = 15) and after BDNF was added in the presence of preapplied U0124 (*n* = 17). **(F)** Mean frequency of MEPPs in control and upon application of BDNF in the presence of U0124. Histograms and error bars represent the mean ± SEM. * *p* < 0.05 compared to control.

It is well known, that TrkB receptors upon activation by BDNF, can trigger the MEK1/2-Erk signaling pathway in neurons [31,32]. To test if this MAPK pathway may take part in modulatory BDNF effects, we used the MEK1/2 inhibitor – U0126 (20 µM). Within 1 h of application, U0126 did not alter neither amplitude nor frequency of MEPPs (Figure 10A). However, when MEK1/2-Erk signaling pathway was blocked, BDNF failed to increase MEPP amplitude, but fully retained the ability to increase the frequency of spontaneously released quanta of ACh (Figure 10C,D). The specificity of MEK1/2 inhibitor was examined using its inactive analog U0124 (20 µM) as a negative control. The application of U0124 was unable to prevent BDNF-induced increase in both amplitude and frequency of MEPPs (Figure 10B,E,F).

**Figure 11:**
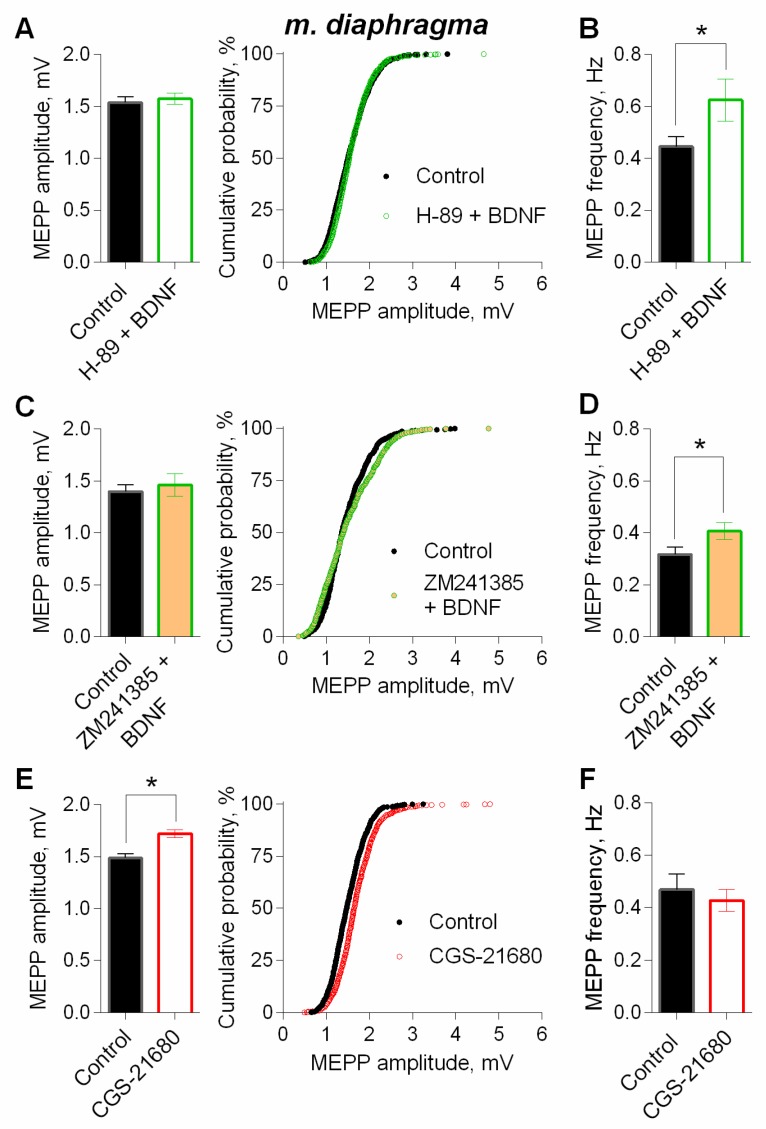
Intracellular signaling mechanisms underlying BDNF-induced upregulation of spontaneous neuromuscular transmission at the mouse diaphragm NMJs. The potentiating effect of BDNF (1 nM) on the amplitude of MEPPs depends on PKA and the activity of adenosine A_2A_ receptors. (**A**) Mean amplitude of MEPPs (left) and cumulative probability plots (right) in control (*n* = 28) and after BDNF was added in the presence of PKA inhibitor H-89 (1 μM) (*n* = 29). (**B**) Mean frequency of MEPPs in control and upon application of BDNF in the presence of H-89. (**C**) Mean amplitude of MEPPs (left) and cumulative probability plots (right) in control (*n* = 19) and after BDNF was added in the presence of A_2A_ receptor antagonist ZM241385 (10 nM) (*n* = 22). (**D**) Mean frequency of MEPPs in control and upon application of BDNF in the presence of ZM241385. (**E**) Mean amplitude of MEPPs (left) and cumulative probability plots (right) in control (*n* = 21) and upon application of A_2A_ receptor agonist CGS21680 (100 nM) (*n* = 29). (**F**) Mean frequency of MEPPs in control and in the presence of CGS21680. Histograms and error bars represent the mean ± SEM. * *p* < 0.05 compared to control.

Since there is evidence about the possible involvement of presynaptic PKA in the regulation of ACh loading into synaptic vesicles at motor synapses [25,33], we further investigated the possible participation of this kinase in BDNF effects. We used H-89 as PKA inhibitor, (1 µM), which did not alter any parameters of MEPPs [25]. We found that inhibition of PKA abolished an increase in the amplitude of MEPPs caused by BDNF, but it was not capable of preventing the neurothrophin-induced increase in MEPP frequency (Figure 11A,B). This allows to suggest that in motor terminals in a resting state (without evoked synaptic activity), there is some tonic activity of PKA, which can, along with the action of BDNF, take part in stimulating of ACh vesicular loading, leading to elevation of quantal size and subsequent increase in the amplitudes of postsynaptic potentials.

PKA activity in motor terminals can be provided by the action of endogenous adenosine on presynaptic G_s_-protein-linked A_2A_ receptors [34,35,36,37]. In this regard, we tested whether the tonic activation of the A_2A_ receptors could maintain the constitutive PKA activity in nerve terminals, and participate in the potentiating effects of BDNF on ACh quantal size.

We applied BDNF on neuromuscular preparations in presence of A_2A_ receptor antagonist ZM241385 (10 nM) or agonist of A_2A_ receptors CGS21680 (100 nM). ZM241385 did not affect the parameters of spontaneous ACh release [37]. In the presence of A_2A_-antagonist, BDNF has lost the ability to significantly increase the amplitude of the MEPPs but was still able to potentiate the MEPP frequency (Figure 11C,D).

Additional stimulation of the A_2A_ receptors by CGS21680 for 90 min led to a statistically significant increase in MEPP amplitude by 18%. In the presence of CGS21680, MEPP frequency did not change in comparison to control (Figure 11E,F).

**Figure 12:**
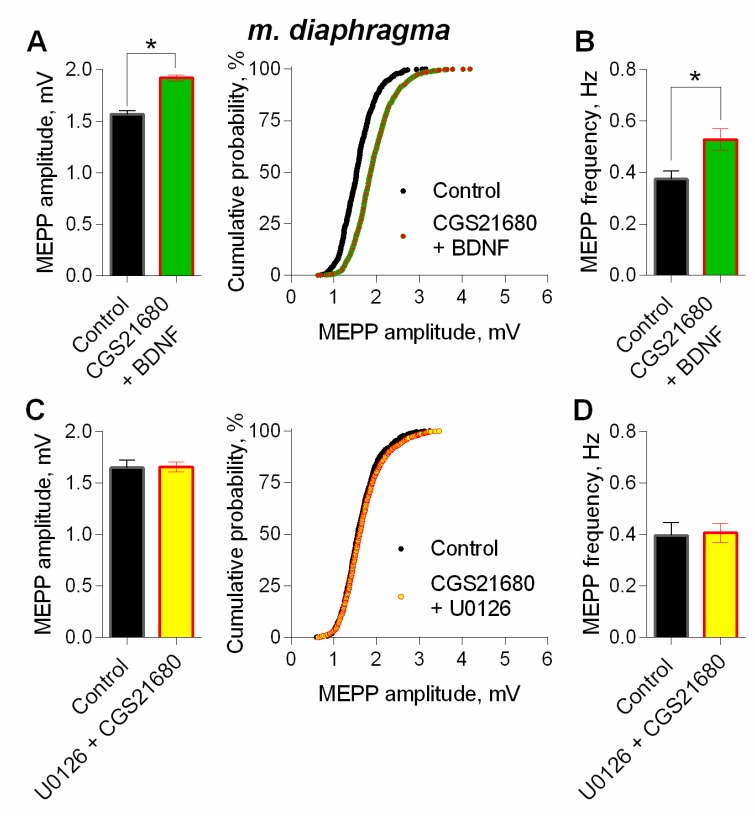
Intracellular signaling mechanisms underlying BDNF-induced upregulation of spontaneous neuromuscular transmission at the mouse diaphragm NMJs. The effect of BDNF (1 nM) on the MEPPs amplitude depends on coupling of A_2A_-receptor-mediated and MEK1/2-mediated intraterminal pathways. (**A**) Mean amplitude of MEPPs (left) and cumulative probability plots (right) in control (*n* = 23) and after BDNF was added during additional stimulation of A_2A_ receptors by their agonist CGS21680 (100 nM) (*n* = 35). (**B**) Mean frequency of MEPPs in control and upon application of BDNF in the presence of CGS21680. (**C**) Mean amplitude of MEPPs (left) and cumulative probability plots (right) in control (*n* = 15) and after CGS21680 (100 nM) was added in the presence of MEK1/2 inhibitor U0126 (20 nM) (*n* = 24). (**D**) Mean frequency of MEPPs in control and upon application of CGS21680 in the presence of U0126. * *p* < 0.05 compared to control.

In order to evaluate the possible additive or occlusive crosstalk between the neurotrophin and A_2A_-mediated effects on spontaneous ACh release, we applied BDNF 30 min after the addition of CGS21680. During stimulation of A_2A_ receptors by CGS216980, BDNF caused an increase in MEPP amplitude, but this BDNF action on MEPPs amplitude was almost undistinguishable from the one obtained during the application of BDNF alone (Figure 12A, see Figure 7A). Therefore, during additional activation of PKA-dependent pathway by A_2A_ receptors stimulation, application of BDNF caused an occlusion of potentiating effects on MEPP amplitude, but not their summation. Application of BDNF in the presence of A_2A_ agonist increased the frequency of MEPPs (Figure 12B).

Finally, to detect whether the activity of A_2A_ receptors is coupled to BDNF-induced MAPK pathway which both lead to an increase in MEPP amplitude, we examined the effect of A_2A_ agonist on spontaneous ACh release when MEK1/2 was inhibited. In the presence of U0126 (20µM) activation of A_2A_ receptors by CGS21680 failed to increase MEPP amplitude (Figure 12C).

Thus, we have established that in mouse motor synapses, the amplitude of MEPPs and EPPs could be upregulated by BDNF in nanomolar concentration. The effect is at least primarily presynaptic and is associated with the enhancement of the ACh loading into synaptic vesicles. This effect of BDNF requires the coupling of two signaling pathways: Firstly, the mandatory participation of the active PKA, supported by the tonic activity of the presynaptic adenosine A_2A_ receptors, and, secondly, BDNF-TrkB-mediated triggering of the MAPK pathway (MEK1/2-mediated) in motor nerve terminals. It is the coupled action of these two signaling pathways that ensures the development of BDNF-induced increase in the ACh quantal size followed by an increase in the amplitude of postsynaptic potentials. Moreover, BDNF accelerated the spontaneous ACh release via PLC-mediated increase in MEPP frequency. BDNF maintained this effect in the presence of either vesamicol or bafilomycin A1, as well as within application of inhibitors of PKA or MAPK signaling pathway. These results suggest that the mechanism underlying the increase in MEPP frequency is mediated by a separate signaling pathway (PLC-dependent), which is not associated with BDNF-induced potentiation of ACh transport to synaptic vesicles. In a similar way, the detected increase in EPP quantal content, induced by BDNF, is apparently mediated by the specific mechanism, which is not related to the one underlying the increase of EPP amplitude due to the upregulation of ACh quantal size.

## 4. Discussion

We are the first to show that low nanomolar concentration of thrombin in the synaptic area of mature or newly-formed mouse motor NMJs can facilitate synaptic transmission due to thrombin-induced increase in EPP amplitude throughout short trains of EPPs. This increase is not accompanied by the growth of EPP quantal content, but instead a parallel increase in MEPPs amplitude by 25–30% occurs. The ability of vesamicol, an inhibitor of vesicular ACh transporter, to prevent the potentiating effect of thrombin provided a strong argument in favor of our suggestion that thrombin can augment the amplitudes of MEPPs and EPPs due to presynaptic enhancement of ACh vesicular transport and increase in quantal size. This novel thrombin activity is in good agreement with the previously described effects of TRAP6, a peptide agonist of PAR1 thrombin receptors, which increased the amplitude of postsynaptic potentials in both mature and newly-formed motor synapses [14]. The similarity between the effects of TRAP6 and thrombin suggests that thrombin-induced modulation of synaptic transmission is mediated by activation of PAR1 receptors, localized on postsynaptic muscle membranes at both newly-formed and mature mouse NMJs [12,15].

Thrombin expression and release from skeletal muscle has been well described [8,9,38]. This serine protease is known to be a regulatory factor of synaptogenesis in embryonic/neonatal muscles and in mature skeletal muscles during denervation-reinnervation plasticity [13,39]. Thrombin activation of muscular PAR1 receptors is believed to trigger downregulation of extrasynaptic nicotinic ACh receptors and accelerate the elimination of redundant synaptic contacts on muscle fibers [8,11,12]. We have established that thrombin in nanomolar concentration also has a novel acute facilitatory effect on synaptic transmission in both the newly-formed and mature NMJs. It is expressed as an increase in the amplitude of spontaneous and evoked postsynaptic potentials without altering the EPP quantal content.

Considering the lack of presynaptic PAR1 receptors at mature and newly-formed NMJs, it can be assumed that thrombin, acting on muscle PAR1 receptors, induces the release of some muscle-derived factor, that provides the presynaptic enhancement of ACh quantal size. Taking into account the discovered ability of TrkB antagonist ANA12 to abolish the potentiating effects of thrombin in mature synapses and our previous data, demonstrating that PAR1 peptide agonist failed to provoke thrombin-like increase in MEPP amplitude when TrkB receptors were blocked [14], it can be implied that myogenic BDNF (as an endogenous TrkB agonist) may serve as such a hypothetical mediator of thrombin action at NMJs.

This assumption is supported by the previous reports demonstrating the expression of BDNF in skeletal muscle [6,7], its release to the synaptic area as a result of electrical and/or contractile muscle activity and subsequent presynaptic TrkB activation [40]. Thrombin, acting on PAR1, may induce BDNF release in platelets [41]. At NMJs, the evidence of functional coupling of thrombin- and BDNF-mediated regulation of MEPP and EPP amplitudes is based not only on the similarity between potentiating effects of thrombin and BDNF, but also on the dependence of these effects on the activity of TrkB and on the action of vesamicol.

The ability of BDNF to affect synaptic transmission in NMJs via activation of presynaptic TrkB [42,43] and to increase the amplitude of postsynaptic potentials and MEPP frequency has been reported previously [26,36,43,44,45]. However, the mechanisms underlying these effects of BDNF remained unclear. The BDNF-induced increase of postsynaptic uniquantal endplate potentials could be of postsynaptic origin in particular due to an increase in input resistance of muscle fibers membrane, or reflect some presynaptic processes increasing the amount of neurotransmitter inside the vesicles. In our work we obtained novel data that the increase in MEPP amplitude induced by exogenous BDNF has a presynaptic origin and is due to the increased vesicular loading of ACh leading to an augmentation of ACh quantal size, since the BDNF effect could be prevented by direct (vesamicol) or indirect (bafilomycin A1) inhibition of ACh vesicular transport at NMJs.

BDNF, acting on TrkB receptors, may trigger the MEK1/2-Erk-mediated signaling pathway in neurons [30,31,32]. The function of this MAPK pathway is usually associated with the regulation of transcription factors followed by gene expression changes. It was suggested that synapsins may serve as a target for presynaptic MEK1/2-Erk signaling. BDNF-induced phosphorylation of synapsins via MAPK pathway stimulates mobilization of synaptic vesicles from the reserve pool to the active zones leading to an increase in the frequency of spontaneous neurotransmitter release in excitatory and inhibitory CNS synapses [46,47,48]. Unlike in the CNS synapses, inhibition of MEK1/2 by U0126 prevents the BDNF-induced increase in MEPP amplitude in NMJs. It is reasonable to suggest that in motor nerve terminals BDNF-triggered MAPK pathway is directed towards stimulation of vesicular ACh transport resulting in an increase in MEPP amplitude. Among the currently known targets of the MEK1/2-Erk-dependent signaling cascade in synapses [49,50] this one (loading of ACh into synaptic vesicles) is new and have not been described yet.

It cannot be excluded, that the vesicular H^+^-ATPase, which is responsible for vesicular acidification and proton-dependent pumping of ACh into the vesicles, could be the hypothesized target of presynaptic MEK1/2-Erk activity at NMJs. This suggestion is supported by the recently discovered structural and functional interaction between the Erk and the V0-subunit of the H^+^-ATPase in late endosomes. Activated Erk provokes stimulation of vacuolar H^+^-ATPase activity and endosomal acidification [51]. Given the common endosomal origin of synaptic vesicular pools [52], it is tempting to assume a similar tight interaction between the BDNF-activated Erk and the vesicular proton pump in motor nerve terminals.

Our data demonstrate that, along with MEK1/2-Erk signaling pathway, the activity of PKA is also necessary for BDNF-induced increase in quantal size, since inhibition of PKA by H-89 prevented the ability of BDNF to augment the amplitude of MEPPs. The requirement of PKA activity for an increase in the amplitude of uniquantal MEPPs due to the enhanced ACh vesicular transport in motor nerve terminals has been reported earlier [25,33,53]. However, the final target of the regulatory action of PKA in these cases remained unclear. Our data and literature reports allow to suggest that activated PKA can interact with MEK1/2-Erk kinases in nerve terminals. Thus, coupling of two signaling pathways seems to be essential for the stimulation of ACh loading into the synaptic vesicles at NMJs. Similar coupling has been described in the CNS synapses, where PKA can interact with the MEK1/2-Erk-mediated signaling pathway triggered by BDNF stimulation of TrkB, although in CNS such interaction was not associated with the modulation of neurotransmitter quantal size [31,54].

In motor nerve terminals a certain level of PKA activity can be maintained via activation of presynaptic G_s_-protein-coupled A_2A_ receptors by endogenous adenosine [34,37]. In the present study we found a novel fact that the blockade of tonic A_2A_ receptors activity results in the loss of BDNF ability to induce an increase in MEPP amplitude at NMJs. BDNF-induced augmentation of MEPP amplitude becomes occluded in the case of preliminary additional stimulation of A_2A_ receptors by their agonist CGS21680, which is able to potentiate the amplitude of MEPPs by itself during prolonged application. Moreover, CGS21680 was unable to increase MEPP amplitude when MAPK pathway was inhibited. Taken together, these data reinforce the idea that BDNF-induced upregulation of ACh quantal size requires the coupling of MAPK pathway with A_2A_ receptors-mediated PKA activity.

Along with MEPP amplitude modulation, we confirmed the ability of BDNF to regulate the frequency of spontaneous quantal ACh release in mature motor synapses. Unexpectedly, we found that this increase in MEPP frequency is mediated by the activation of PLC-dependent phosphoinositide pathway, which can also be triggered by BDNF, but separately and independently from the MAPK pathway. The BDNF/TrkB/PLC-dependent signaling followed by subsequent release of stored calcium and protein kinase C activation is known to modulate evoked neurotransmitter release in CNS synapses [55]. The ability of BDNF to rapidly accelerate spontaneous release by acting on TrkB, was also demonstrated in central synapses. Among the mechanisms of this effect, the activation of Erk-mediated MAPK pathway followed by phosphorylation of synapsins, the activation of Ca^2+^-permeable presynaptic TRPC channels or the release of stored calcium are suggested [48,56]. Interestingly, BDNF-driven acceleration of spontaneous neurotransmitter secretion in CNS is often A_2A_-dependent [57,58]. Increase in the frequency of spontaneous ACh quantal secretion through BDNF-induced activation of presynaptic PLC has not been demonstrated earlier in motor synapses.

A comparative analysis has shown the inability of thrombin (as possible activator of muscle-derived endogenous BDNF release) to increase the MEPP frequency both in mature and newly-formed NMJs, while the exogenously applied BDNF augments this parameter of spontaneous ACh release in mature motor synapses. Such variability and ambiguity of the effects triggered by exogenous versus endogenous (thrombin-initiated) neurotrophin action may be due to many factors, including the differences in effective concentrations of endogenously released BDNF and proBDNF in comparison to exogenously applied mature form of neurothrophin [55], a complex turnover of endogenous BDNF and its receptors (TrkB and p75) at synaptic terminals [59], functional crosstalk between TrkB and other presynaptic receptors [26,43], and relative contribution of different BDNF sources during various patterns of synaptic activity.

## 5. Conclusions

In summary, our comparative analysis of thrombin and BDNF potentiating effects in mouse NMJs showed that both factors act similarly on neuromuscular synaptic transmission, inducing an enhancement of vesicular ACh loading and subsequent increase in ACh quantal size. We have also found evidence in favor of possible role of BDNF as a novel agent mediating thrombin-induced signaling in motor synapses. This allows to suggest more extensive homeostatic role of this serine protease and its receptors in neuromuscular system. Despite regular discussions and vast amounts of research [60,61,62], the conditions leading to such non-coagulative activity of endogenous thrombin in various tissues, and in mature muscles particularly, remain largely unknown. The expression of PAR1 receptors and thrombin and their regulatory activity have already been identified in immature (embryonic and neonatal) [39] or in mature skeletal muscles during denervation-reinnervation plasticity, inflammatory myopathies or injury [63,64].

**Figure 13:**
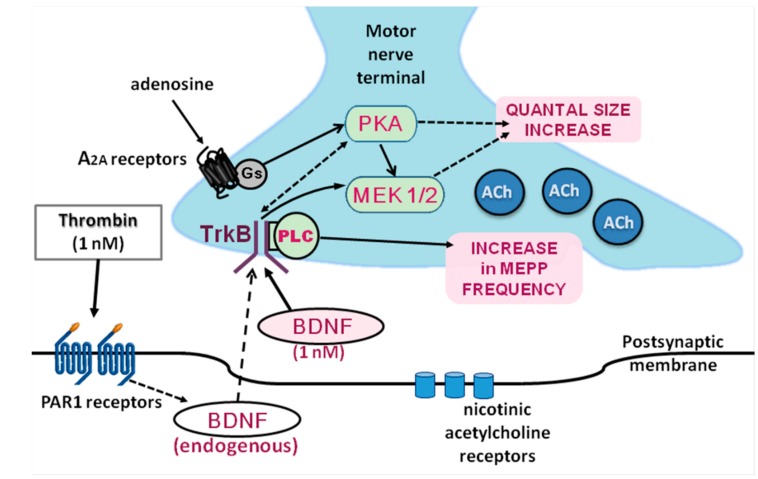
Suggested signaling pathways underlying the potentiating action of thrombin and BDNF at NMJs.

Unlike thrombin, the release of BDNF from skeletal muscles and its further local effects in skeletal muscles and NMJs were studied more [4,42,65]. In our work, some novel targets and signaling mechanisms of BDNF regulatory action in NMJs have been suggested (Figure 13). They expand our understanding of the functional activity of thrombin and BDNF in different types of synaptic contacts.

Thus, the revealed acute thrombin and BDNF modulatory activity aimed towards potentiation of quantal ACh secretion at NMJs suggests, that both factors, if being secreted from muscle fibers, may act as effective regulators maintaining a high level of the neuromuscular transmission safety factor. The particular conditions of triggering and further realization of this modulatory activity require more detailed investigation.
